# Digital Innovation in Asthma Management in Italy: Results From the “Confronting Asthma Survey”

**DOI:** 10.1002/clt2.70109

**Published:** 2025-10-17

**Authors:** Manuela Latorre, Pierluigi Paggiaro, Cristina Cardini, Giovanni Paoletti, Emanuele Nappi, Mario Di Gioacchino, Enrico Heffler, Francesco Blasi, Giorgio Walter Canonica, Francesca Puggioni, SANI Network

**Affiliations:** ^1^ Pulmonary Unit Nuovo Ospedale Apuano Massa Italy; ^2^ Department of Surgery, Medicine, Molecular Biology and Critical Care University of Pisa Pisa Italy; ^3^ Fondazione per la Salute Respiratoria della Società Italiana di Pneumologia SIP‐IRS Milan Italy; ^4^ Department of Biomedical Sciences Humanitas University Milan Italy; ^5^ Personalized Medicine Asthma and Allergy IRCCS Humanitas Research Hospital Rozzano Italy; ^6^ Center for Advanced Studies and Technology (CAST) G. D'Annunzio University of Chieti‐Pescara Pescara Italy; ^7^ Institute of Clinical Immunotherapy and Advanced Biological Treatments Pescara Italy; ^8^ Respiratory Unit and Cystic Fibrosis Center Fondazione IRCCS Ca’ Granda Ospedale Maggiore Policlinico di Milano Milan Italy; ^9^ Department of Pathophysiology and Transplantation University of Milan Milan Italy

**Keywords:** asthma management, barriers, digital innovation, eHealth, knowledge gap

## Abstract

**Background:**

The rapid digital transformation has significantly impacted healthcare, particularly through eHealth solutions that offer great potential for managing chronic respiratory conditions such as asthma. This study explores the impact, attitudes, and acceptance of digital technologies in asthma management in Italy.

**Methods:**

A structured 34‐item survey, developed in two versions for patients and physicians, was administered independently and anonymously to adult asthma patients and to specialists in pulmonology and allergology. The questionnaires collected data on demographics, professional background, digital habits (for work and leisure), use of digital tools such as apps and smart inhalers, doctor–patient digital communication, familiarity with online health resources, and perceived barriers to digital adoption. Data were collected anonymously via REDCap, with oversight from the Severe Asthma Network in Italy (SANI).

**Results:**

A total of 134 patients and 180 doctors participated. Findings revealed a predominantly positive attitude toward digital tools, with 85% of physicians and 74.4% of patients embracing a “digital mindset.” Nevertheless, digital innovations remain underutilized in clinical practice. While 85.6% of patients reported regularly using digital tools in their daily lives, 91.5% stated that their doctors had never recommended apps or websites for asthma self‐management. Digital solutions such as mobile apps, wearable spirometers, and telemedicine are recognized for their potential benefits—clinicians highlighted symptoms self‐tracking (17.2%), improved adherence (22.7%), and enhanced clinical interventions (11.7%) as key advantages. However, adoption is hindered by concerns such as information technology (IT) compliance (62.5%), legal risks (11.5%), and skepticism about the reliability of remote data (40.6%). Furthermore, 59.9% of clinicians and 66.8% of patients recognized a knowledge gap regarding the potential benefits of smart inhalers and digital therapeutics in respiratory care.

**Conclusion:**

The study highlights a positive attitude toward digital tools in asthma management but reveals limited adoption in clinical practice. Key barriers include IT compliance concerns and knowledge gaps. Addressing these challenges through education and regulatory support could enhance digital integration, improving asthma care.

## 1 Background

Healthcare has not been exempt from the rapid digital transformation that has deeply influenced our daily lives, habits, behaviors, and overall quality of life. The COVID‐19 pandemic significantly accelerated the adoption of digital communication and tools in healthcare.

The World Health Organization defines ‘eHealth’ as the use of information and communication technologies for health [1]. This includes a wide range of technologies and tools such as mobile health (mHealth), telemedicine, social media platforms, and health informatics like electronic health records (EHRs) and clinical decision support systems, all aimed at improving the efficiency and effectiveness of healthcare [2, 3].

Healthcare digitalization across Europe is driven by strategic initiatives at both EU and national levels. The Next Generation EU/PNRR (Italy) 2021–2026 program supports the digitalization of healthcare, focusing on initiatives such as e‐prescriptions, electronic health records (EHRs), and telemedicine. Table 1 highlights key initiatives from the EU and Italy.

**TABLE 1:** Key EU digital‐health policies and Italian national initiatives driving healthcare digitalization.

Initiative	Scope and timeline	Role in digitalization
EU4Health program (2021–2027) [4]	EU‐wide; 2021–2027	Strengthens health systems, supports digital tools, telemedicine, and cross‐border data exchange
European health data space (EHDS) [5]	EU‐wide; from 2025	The EHDS aims to enable secure access and reuse of health data across the EU for care and research, addressing barriers revealed during COVID‐19 while raising legal and ethical challenges
Health data ecosystem and electronic health records 2.0 (Italy) [6, 7]	Decree sept 2023; operational by March 2026	Central platform for health data, providing patient access, standardized digital records and data‐sharing aligned with EU/EHDS goals
National telemedicine platform (Italy) [8]	Italy; launched 2023	Integrates and standardizes regional telemedicine services for nationwide coverage

Digital healthcare solutions have the potential to meet the treatment needs of chronic respiratory disorders such as bronchial asthma [9].

In particular, adopting technology could be highly beneficial in managing asthma, which affects a large portion of the population, including many young people who need effective self‐management and patient empowerment. Despite significant advancements in personal technology use, healthcare has needed to integrate these innovations faster, and more research is required to identify the most effective interventions for different patients.

Various eHealth tools, such as asthma apps, online health diaries, and app‐based portable spirometers, have demonstrated the potential to improve asthma management [10, 11]. Artificial intelligence (AI) programs designed for patient education and advanced counseling also contribute to this goal [12].

However, using digital health technologies to manage respiratory and allergic diseases like asthma presents several challenges. These include issues of accessibility to digital healthcare systems and the varying digital skills of both doctors and patients. Overcoming these obstacles requires further clarification and research to ensure these technologies can be effectively and equitably implemented.

In this context, we aimed at exploring the impact, attitudes, and acceptance of digital innovations in the management and treatment of asthma in Italy, focusing on the perspectives of both healthcare professionals and asthma patients, through the use of a specially designed survey developed by the Severe Asthma Network in Italy (SANI) group [13].

## 2 Methods

This cross‐sectional, observational study aimed to explore the perspectives, attitudes, and behaviors of physicians and asthmatic patients toward digital innovation in asthma care across Italy. To achieve this, a nationwide survey methodology was selected as the most appropriate and effective approach, given its ability to systematically collect standardized data from predefined populations.

### 2.1 Study Design and Participants

The study population included adult asthma patients and medical specialists in pulmonology and allergology. A purposive sampling strategy was employed to ensure broad representation across geographical regions (North, Central, and South Italy), clinical specialties, and educational backgrounds among patients. This strategic selection ensured a diverse pool of participants and mitigated the risk that only individuals with higher digital literacy or greater familiarity with digital health would respond to the survey.

Two structured questionnaires—one for patients and one for physicians—were developed, each consisting of 34 items that included closed‐ended, Likert‐scale, and open‐ended questions. The instruments were designed to assess demographic and professional background, digital habits (both for work and leisure), use and management of digital tools (e.g., apps, smart inhalers), modes and frequency of digital doctor–patient communication, familiarity with online health resources and perceived barriers to the adoption of digital health tools.

The questionnaires were developed by a multidisciplinary expert panel within the Severe Asthma Network in Italy (SANI) to ensure clarity, neutrality, and alignment with the study's objectives, thereby reducing the risk of information bias.

The initial version of the questionnaire was tested on a small sample of asthmatic patients to evaluate the clarity, comprehensibility, and overall quality of the items. Based on feedback from this pilot phase, the final version was refined and supplemented with definitions of key terms—such as *digital mindset*, *smart inhaler*, and *digital therapeutics*—to improve participants' understanding.

The survey was administered using REDCap (Research Electronic Data Capture), a secure, web‐based platform that ensured standardized, anonymous, and independent completion by both patients and physicians. This helped reduce interviewer and response bias while maintaining data integrity and participant confidentiality.

The physicians involved in the study were recruited from the SANI network, which includes specialized centers for the treatment of severe asthma throughout Italy and operates in close collaboration with major national scientific societies. Clinicians were selected across different regions of the country—including the North, Center, and South—and were trained to enroll patients from diverse educational backgrounds. These physicians also completed their own version of the survey, aimed at capturing their perspectives on digital health tools and practices in asthma care.

Informed consent was obtained electronically from all participants prior to survey initiation.

This study was conducted under the SANI observational study protocol, approved by the Local Ethical Committee of Area Vasta NORD‐OVEST Toscana (Protocol number 73714, December 2016), and was retrospectively registered on ClinicalTrials.gov (ID NCT06625216; October 2024).

### 2.2 Data Analysis

Two hundred forty clinicians from the SANI network were invited to participate in the study, and 180 of them—representing 75.0% of those invited—were enrolled, having completed at least 50% of the questionnaire. Additionally, 154 patients were recruited during routine clinical visits. Although no formal sample size calculation was performed due to the exploratory nature of the research, the sample size was considered sufficient for generating meaningful descriptive insights. It was designed to reflect a balance between feasibility and representativeness, allowing the identification of trends and barriers in digital health implementation.

Data were analyzed using descriptive statistics. Results for continuous variables were reported as mean ± standard deviation, while nominal variables were presented as absolute frequencies and percentages. This analytic approach adhered to best practices for cross‐sectional survey research to ensure clarity, reproducibility, and comparability. Group comparisons were conducted using Chi‐squared test and two‐tailed *p*‐value < 0.05 was considered statistically significant. Statistical analyses were performed using SAS statistical software version 9.4.

## 3 Results

**FIGURE 1:**
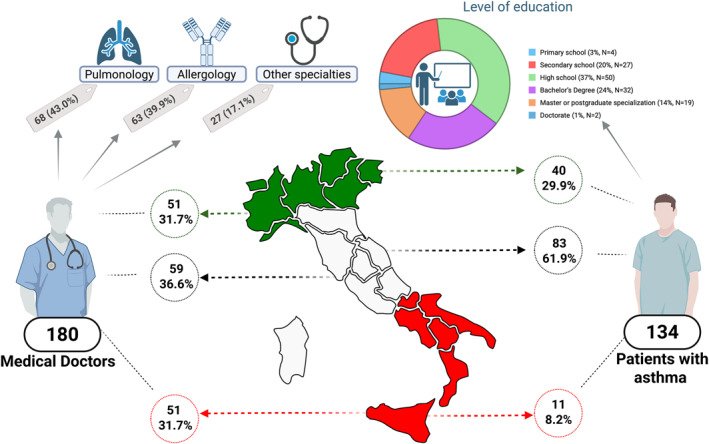
Summary of the educational background and geographical distribution of the survey respondents, categorized into Medical Doctors and Patients with asthma. Responses from both groups are reported as absolute frequencies and percentages. Most physician respondents were specialists in Allergology or Pulmonology; however, the survey also included a smaller proportion of other medical professionals who are routinely involved in asthma care. In the upper‐right corner of the figure, a pie chart illustrates the educational attainment of patient respondents, revealing that the vast majority had completed at least a high school education. Created with BioRender.com.

**FIGURE 2:**
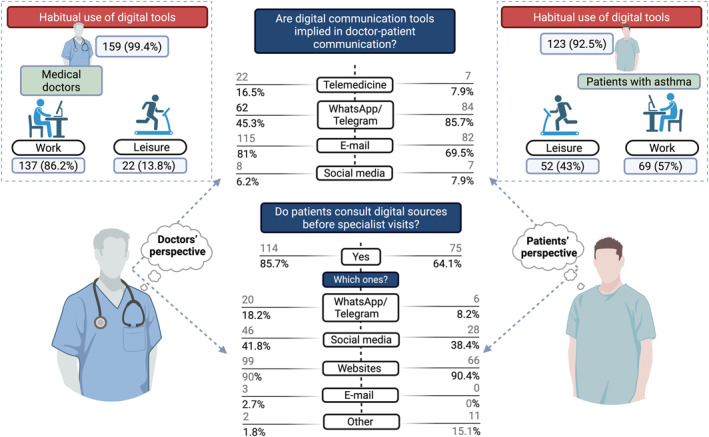
Comparative overview of the attitudes of Medical Doctors and patients with asthma toward digital technologies. The responses are visually divided, with patients' perspectives displayed on the right and doctors' on the left. Initially, participants were asked whether they regularly use digital devices in their daily lives, and then to specify if for professional purposes or leisure activities. Subsequent questions explored their views on the integration of digital communication tools into clinical practice. Responses from both groups are reported as absolute frequencies and percentages, positioned respectively on either side of each item under evaluation. Created by BioRender.com.

In total, 134 asthmatic patients and 180 doctors participated in the survey. The Figures 1 and 2 summarize the main characteristics and attitudes of doctors and patients toward digital technology.

### 3.1 Population Characteristics

53.7% of the surveyed patients were > 50 years old, with a significant majority being women (70.9%). The median duration of asthma among these patients was 10–15 years, and 80.8% reported having moderate or severe asthma. Note that the degree of asthma severity was patient‐reported, implying that it is not necessary in line with current definitions of asthma severity.

While 54.1% of surveyed doctors worked in general hospitals, 45.9% worked in university hospitals, and 14.0% worked in local clinics widespread on the national territory (Figure 1).

Notably, 85.0% of physicians and 74.4% of patients expressed a positive attitude toward digital technology, indicating that they either fully or somewhat embraced a digital mindset.

One hundred sixteen surveyed physicians (64.4%) stated that digital technology could improve their practice, and 51 responders (28.3%) reported implementing digital innovation in their ambulatories.

However, the use of telemedicine, wearables, and smart inhalers was relatively low and did not differ significantly across specialties. Nevertheless, allergologists were significantly more likely to recommend health‐promoting apps compared to pulmonologists (41.3% vs. 25.0%, *p* = 0.02) (Supporting Information S3: Table 2S).

The majority (85.6%) of surveyed patients reported regularly using digital tools in at least one of various activities in their daily lives, including at work, at home, at school, and in their relationships.

Among asthmatic patients, the use of digital tools decreased with age, with 100% of patients under 30 years using them habitually, compared to 96.1% in the 30–50 group and 84.7% in those over 50.

Telemedicine use and awareness or use of smart inhalers were low across all age groups. Younger patients showed a higher tendency to use wearables and recommended health‐promoting apps, though these differences also lacked statistical significance (Supporting Information S3: Table 2S).

Furthermore, when surveyed on questions referring to digital health initiatives in general—regardless of disease area and not limited to asthma‐specific interventions—56 physicians (38.6%) reported that their organization had promoted initiatives specifically related to digital health. The most frequently implemented initiatives were the adoption of digital tools to support medical practice (40 physicians; 71.4%), followed by educational initiatives (19; 33.9%), digital tools aimed at supporting patients (15; 26.8%), and research activities (11; 19.6%).

Regarding satisfaction with these initiatives, 15 physicians (26.8%) described them as completely satisfactory, while 30 (53.6%) found them fairly satisfactory. A smaller proportion expressed uncertainty (8; 14.3%) or dissatisfaction—2 doctor (3.6%) were rather dissatisfied and 1 (1.8%) completely dissatisfied.

Among the 125 patient respondents to these questions, 85 (68.0%) indicated that they had recently been encouraged or had more opportunities to use digital tools for managing their health. The most commonly promoted tools or initiatives included informational resources related to disease or disease management (69 patients; 81.2%), digital tools enabling physician support (43; 50.6%), tools for clinical parameter monitoring (12; 14.1%), and telemedicine visits (6; 7.1%).

Patient satisfaction with these tools was high: 34 respondents (40.0%) rated their experience as completely satisfactory and 45 (52.9%) as fairly satisfactory.

### 3.2 Perceptions, Use, and Barriers to Digital Health Communication and Telemedicine

Figure 2 presents an overview of the digital communication tools utilized between clinicians and patients. A substantial proportion of clinicians reported concerns regarding the legal implications of employing various digital platforms—such as email, messaging applications (e.g., WhatsApp, Telegram), and social media platforms (e.g., Facebook, Instagram)—for professional communication. Specifically, 73.8% of clinicians expressed concern about the use of email, 86.4% regarding WhatsApp or Telegram, and 98.6% concerning the use of websites and social media channels.

In contrast, patients demonstrated comparatively lower levels of concern about these legal aspects. Among respondents, 42.3% expressed apprehension about email communication, 48.3% about the use of messaging applications, and only 22.9% reported concern over the use of websites and social media for interacting with healthcare providers.

Regarding digital apps and tools, 36.3% of the doctors expected patients to use health‐promoting digital apps like Allergy Diary and apps for self‐monitoring asthma and allergy control.

Clinicians rated the benefits of digital tools as symptom self‐tracking (17.2%), support for clinical interventions (11.7%), improved adherence (22.7%), and the collection of clinical data for real‐life studies (9.7%).

Despite these potential benefits, 91.5% of patients reported that their doctors had never recommended using apps or websites for better asthma self‐management.

However, the asthmatic patients surveyed considered these tools potentially useful for various purposes: 50.0% believed they could improve awareness and knowledge of their disease, 37.5% thought they could be used to share data with their doctor to assess the degree of disease control, and another 37.5% found them helpful for remembering to take their therapy.

According to 85.7% of doctors, patients independently searched for information on internet websites before visiting the doctor; 64.1% of the patients surveyed confirmed this. Details about online sources consulted by patients are reported in Figure 2.

Telemedicine was perceived as both an opportunity and a challenge within the clinical setting. Among participating clinicians, 22.0% reported using telemedicine for follow‐up visits, whereas only 5.3% considered it suitable for initial consultations. Furthermore, 20.1% of clinicians reported frequent use of digital health technologies, including wearable devices, smart spirometers, and smart oximeters.

However, several barriers to the broader adoption of telemedicine were identified. The most frequently reported obstacles included IT compliance issues (62.5%), medico‐legal concerns (11.5%), and a lack of confidence in remotely collected data compared to that obtained during in‐person visits (40.6%).

From the patient perspective, 91.9% indicated they did not routinely engage in telemedicine. The predominant reason cited was the absence of telemedicine service offerings from their healthcare providers, reported by 80.0% of these patients.

### 3.3 Smart Inhalers and Digital Therapeutics

Awareness and use of smart inhalers were also examined.

Concerning the potential benefits of smart inhalers, according to surveyed doctors, they offer several significant benefits for patients. Most clinicians (71.5%) believe that smart inhalers can help patients remember to take their treatment regularly, and 60.8% think that these devices can assist in improving patients' inhalation techniques. Furthermore, 46.9% of doctors consider smart inhalers beneficial in preventing and managing asthma exacerbations. On the other hand, patient awareness of smart inhalers is relatively low: 41.1% of patients were aware of smart inhalers but only 5.4% used them, while 58.9% of patients were unaware of their existence.

With respect to the awareness of digital therapeutics, doctors and patients admitted a significant knowledge gap regarding the importance and potential benefits of digital therapeutics (DTx) in respiratory diseases. Specifically, 59.9% of clinicians and 66.8% of patients believed there needs to be more knowledge about the importance and potential benefits of DTx in managing respiratory conditions.

## 4 Discussion

This study provides a comprehensive analysis of the impact, acceptance, and attitudes toward digital innovation in asthma management among patients and healthcare professionals in Italy. Using a nationwide survey methodology, we gathered perspectives from pulmonologists, allergists, and asthmatic patients, with higher response rates observed in the central and northern regions of the country. The decision to adopt a structured survey design was guided by the objective of systematically exploring behaviors, experiences, and perceptions related to digital health tools. This approach enabled the collection of standardized data across diverse geographic and professional contexts, capturing key variables such as digital communication practices, familiarity with smart inhalers, perceived barriers to adoption, and sources of online medical information.

The primary focus of our survey was on assessing the prevalence of a “digital mindset,” defined as an open‐minded attitude toward the opportunities presented by digital technologies, including incorporating these tools into daily life and professional practices [14].

Most doctors and patients reported having a digital mindset, reflecting broad acceptance and routine use of digital tools. For healthcare professionals, it means integrating these tools into clinical practice, while for patients, it implies using them for better asthma self‐management and communication with doctors [15].

The World Health Organization (WHO) acknowledges the significant role that digital technologies can play in strengthening health systems [16]. The shift toward digital health, which includes eHealth, telehealth, and mobile health (mHealth), can improve the management of chronic conditions like asthma, enhance patient outcomes, and create a more efficient healthcare system [16, 17].

The results of our survey demonstrate that both physicians and patients report increased opportunities or encouragement to use digital tools for general health management. However, when focusing specifically on asthma care, a noticeable gap emerges between the broader availability of digital health solutions and their disease‐specific implementation. This discrepancy suggests that while digital health is gaining ground system‐wide, targeted integration within asthma management remains limited and requires structured action.

One key barrier appears to be limited professional training. Only 33.9% of physicians in our survey reported having access to digital health education. To address clinicians' concerns regarding IT compliance, targeted Continuing Medical Education (CME) modules should be developed to strengthen knowledge of data privacy, security, and regulatory requirements related to digital and AI tools [18]. In addition, institutional training initiatives, such as workshops or online modules, can provide hands‐on guidance through real‐world case studies, enabling practitioners to identify potential risks (e.g., data breaches or ethical concerns) and implement appropriate safeguards. Embedding these measures into professional development pathways would enhance clinicians' confidence in adopting new technologies while ensuring alignment with IT compliance standards [19]. Another area requiring attention is access to home‐based monitoring technologies. While remote spirometry and wearable devices can enable timely detection of respiratory deterioration and reduce unnecessary clinical visits, their uptake among patients in our survey was only 14.1%. Collaborative initiatives involving regional health authorities, technology developers, and academic institutions could support the subsidization of such projects, particularly in underserved regions, and contribute to evaluate their feasibility and cost‐effectiveness.

Additionally, the strong interest in digital health tools among patients (with over 80% expressing interest in disease management resources) underscores the need for co‐designed digital literacy and engagement programs. These should include mobile applications for symptom tracking, AI‐based triage assistants, and tools that offer real‐time, personalized feedback. Proven models such as the UK's *myCOPD* platform highlight the clinical and operational benefits of these approaches [20].

Our survey also identified several perceived benefits of digital tools, such as mobile apps, wearable devices, and telemedicine platforms. These technologies could be crucial in monitoring symptoms, tracking medication use, and facilitating communication between patients and doctors [21, 22]. However, only a few doctors have implemented these innovations in their practices, highlighting a gap between acceptance and actual implementation.

Asthmatic patients surveyed believe these tools can improve disease awareness and self‐management, help share data with doctors, and remind them of their therapy schedules. This is in line with previous literature data confirming the multifaceted advantages of digital tools in patient education, data sharing, and adherence support [23].

An interesting point from our survey is the change in communication methods between doctors and patients, with email and WhatsApp preferred over telemedicine or social media [24, 25].

Although these results suggest shifting attitudes toward digital communication, it is important to point out that they represent perceived preferences rather than confirmed changes in actual clinical practice. Further observational data would be needed to substantiate these findings.

However, telemedicine is increasingly used to improve asthma health outcomes.

In a meta‐analysis that included 22 studies with 10,281 participants, combined telemedicine interventions—such as tele‐case management and tele‐consultation—effectively improved asthma control and quality of life in adults [26].

Despite their potential benefits, significant challenges persist in using digital tools in real‐world clinical practice [27]. This is partly due to inconsistent evidence from some clinical trials regarding their effectiveness in improving asthma control and clinical outcomes.

A systematic review up to 2016 identified various features in mobile apps that support asthma self‐management, adherence, and clinical effectiveness. However, the impact of these features on health‐related outcomes was limited [28]. Another review of app effects on asthma self‐management found some improvements in asthma control, lung function, and quality of life, but no significant impact on self‐efficacy was observed [29]. More recent studies have shown promising results: a randomized controlled trial involving 60 patients demonstrated that a smartphone‐based self‐management app led to significantly greater improvements in asthma control and quality of life at 6 months compared to usual care (*p* < 0.001) [30]. Similarly, a usability study of the PEAK‐mAAP app for adolescents reported a high mean usability score (83/100), indicating strong acceptance, with older adolescents rating the app even more favorably. These findings suggest that well‐designed, user‐centered digital interventions can positively affect both engagement and clinical outcomes, though further large‐scale evaluations are needed [31]. However, in our survey, 91.5% of patients reported that their physician had never recommended the use of apps or websites to support asthma self‐management. This represents a significant barrier to the adoption of digital tools, as more recent extensions of the Technology Acceptance Model have identified physician recommendation as a key social factor influencing technology acceptance. In particular, the doctor–patient relationship plays a critical role in shaping behavioral intention and actual use of digital health technologies [32]. Some studies have shown that trust in the healthcare provider, and the explicit endorsement of digital tools, significantly increase patient willingness to engage with mobile health applications [33].

This potential impact may be undermined, however, by clinicians' uncertainty regarding legal responsibilities, as reflected in our survey, which revealed concerns—particularly about the use of social media and digital tools in professional settings.

These concerns have become more pronounced in the post‐pandemic era. To mitigate legal risks and ensure strong data protection, it is essential to maintain strict patient confidentiality and adhere to high standards of healthcare delivery [14]. In this context, recent national guidelines in Italy have defined a unified framework for telemedicine reimbursement, ensuring services are regulated, clinically appropriate, and financially supported when integrated with electronic health records [30]. Data Protection Officers (DPOs) play a critical role by guiding healthcare providers in compliance with the General Data Protection Regulation (GDPR), the EU's legal framework for personal data protection. They help ensure secure data processing, informed consent, and risk management—key elements in promoting trust and responsible use of digital health tools [34].

Additionally, surveyed doctors noted that some hospital facilities needed to be adequately equipped for digital practices. Patients also reported similar challenges, mentioning difficulties in using the technology and a lack of access to necessary devices or internet connectivity. WHO's Global Digital Health Strategy 2020–2025 aims to strengthen health systems worldwide by integrating digital health technologies, underlining the importance of international cooperation to harmonize regulatory frameworks and ensure equitable access to digital health benefits [16]. However, personalized interventions are still needed to reduce inequalities in healthcare, as telemedicine can sometimes pose a barrier for people without the necessary devices, internet access, or digital skills. Patient associations can also play an important role in supporting digital literacy, and several initiatives aimed at helping asthmatic and COPD patients acquire such skills have already been implemented in Italy.

Finally, this survey explored the awareness and acceptance of using smart inhalers and DTx among doctors and patients. Smart inhalers, which electronically monitor medication usage events, and DTx, which provides therapeutic interventions through software, are emerging technologies that can potentially improve asthma management [35].

Smart inhalers, connected to smartphones, are promising tools that can provide information about the patient's adherence and the inhaler technique [36].

Some studies have explored asthma patients' perceptions of electronic inhaler reminders designed to improve medication adherence. These studies found that such reminders are generally acceptable and effective in helping patients adhere to their treatment and manage their asthma better [37]. However, some patients may still need ongoing support [38].

DTx have demonstrated considerable benefits in managing respiratory diseases, providing innovative solutions that enhance patient outcomes and healthcare delivery.

Particularly, EASYBREATH, a digital therapeutic tool designed for pulmonary rehabilitation, has been demonstrated to improve exercise capacity, alleviated dyspnea, and enhanced the overall quality of life for patients with chronic obstructive respiratory diseases over 8 weeks [39]. Moreover, digital therapeutics enable continuous monitoring of key respiratory biomarkers and vital signs, both in clinical and home settings therefore, in asthma care, it could be useful for empowering patients to manage their conditions better, improving treatment adherence, and enabling the choice for personalized treatment plans [40].

Unfortunately, smart inhalers and DTx are unavailable in Italy for the treatment of asthma. The main limitations in disseminating digital therapeutics and smart inhalers include challenges related to integrating these advanced technologies into routine healthcare practices.

This could be due to various factors, including technological, regulatory, and infrastructural barriers specific to the Italian healthcare system [40].

Interestingly, a qualitative study conducted in the Netherlands by van de Hei et al. found similar issues. Despite interest among stakeholders, most participants (including patients and healthcare providers) had no direct experience using smart inhalers. The study identified key barriers such as lack of reimbursement policies, insufficient evidence on clinical effectiveness, poor integration with electronic health records, and concerns over data privacy. These findings suggest that adoption challenges are not unique to Italy but are shared across multiple European countries [41].

The existing literature needs to provide more extensive and clear data specifically addressing the challenges in Italy regarding disseminating digital tools and technology in healthcare settings. This indicates a potential gap and highlights the need for more localized research on this topic. Our study aims to take a small step toward filling this gap, and it represents a starting point for planning new strategies to address the current limitations of adopting digital technologies in the medical field.

This study has some limitations. The patient survey yielded limited responses to certain questions and showed an unequal distribution, with Southern Italy being underrepresented. To address this issue in future research, oversampling or applying statistical weighting for underrepresented areas should be considered.

In addition, the absence of a defined sampling frame for patient recruitment prevented calculation of an exact response rate, limiting the ability to fully assess selection bias. Nevertheless, the purposive sampling strategy used across diverse clinical settings and regions, combined with an adequate overall sample size, supports the generalizability of our findings. Moreover, no internal reliability metric was calculated for the attitude scales; however, the questionnaire underwent cognitive testing with a small sample of asthma patients to assess clarity and comprehensibility. This approach was considered appropriate given the exploratory nature of the study, although these limitations should be taken into account when interpreting the results and drawing conclusions.

Despite these limitations, our survey offers a realistic snapshot of current attitudes and practices regarding digital innovation in asthma care in Italy. Gathering even more data could be a future objective.

In conclusion, recent research further corroborates the findings of our survey, emphasizing the growing importance of digital tools in respiratory disease management. Bonini et al. highlighted that eHealth interventions, including mHealth apps, telemedicine, and electronic health records, have shown potential to enhance asthma control and patient adherence, despite the current evidence being of moderate quality and marked heterogeneity in study endpoints [42]. Similarly, the MASK‐air study by Sousa‐Pinto et al. underscored the utility of mHealth applications in capturing real‐world data on allergic rhinitis and asthma multimorbidity, revealing that asthma is more strongly associated with persistent rhinitis than its severity. The presence of asthma correlated with poorer rhinitis control [43].

The survey highlights a high level of digital mindset among both groups, indicating openness to digital health innovations. However, barriers such as digital literacy, legal concerns, and limited adoption of advanced digital tools must be addressed [44]. Enhancing education on digital therapeutics and expanding access to digital tools could significantly improve asthma management and overall healthcare quality in Italy. Targeted efforts are needed to bridge the gap between acceptance and implementation of digital tools, allowing healthcare providers to better leverage these innovations for improved patient outcomes.

## Author Contributions

**Manuela Latorre:** conceptualization, investigation, writing – original draft, methodology, validation, writing – review and editing, project administration, supervision. **Pierluigi Paggiaro:** conceptualization, funding acquisition. **Cristina Cardini:** software, formal analysis, data curation, resources. **Giovanni Paoletti:** visualization. **Emanuele Nappi:** Writing – review and editing. **Mario Di Gioacchino:** writing – review & editing. **Enrico Heffler:** writing – review and editing. **Francesco Blasi:** writing – review and editing. **Giorgio Walter Canonica:** funding acquisition, investigation. **Francesca Puggioni:** conceptualization, investigation, methodology, validation, project administration, writing – review and editing.

## Conflicts of Interest

The authors declare that they do not have any potential conflicts of interest in relation to any aspect of this work.

## Supporting information


Supporting Information S1

Supporting Information S2

Supporting Information S3

## Data Availability

The data that support the findings of this study are available from the corresponding author upon reasonable request.
