# Supplementary material for: Digital Innovation in Asthma Management in Italy: Results From the “Confronting Asthma Survey”
Source: Clin Transl Allergy. 2025 Oct 17;15(10):e70109. doi: 10.1002/clt2.70109 (PMC12533498; doi:10.1002/clt2.70109)
Supplement: Supplementary file 1 — Supporting Information S1 [file CLT2-15-e70109-s003.docx]

# DIGITAL INNOVATION SURVEY – FOR PATIENTS

Dear participant,

We thank you in advance for your availability and interest in this research, which aims to gather information about knowledge and attitudes towards the use of digital health tools in the healthcare field.

Our goal is to describe the technological means used by asthmatic patients and doctors to communicate, clarify the types of content they exchange, and obtain an evaluation of the quality and effectiveness of digital communication.

The completion of the questionnaire will take approximately 10 minutes, but you are free to discontinue at any moment.

We guarantee that all collected data will be treated with the utmost confidentiality and privacy, will be analyzed anonymously, and used exclusively for research purposes.

By selecting "Confirm," you declare that you have understood the purpose of the study and authorize the processing of personal data for research activities in anonymous form

- Confirm
- Do not confirm

## Section 1

- You are:
- Male
- Female
- In which region do you live?
- Abruzzo
- Basilicata
- Calabria
- Campania
- Emilia Romagna
- Friuli Venezia Giulia
- Lazio
- Liguria
- Lombardy
- Marche
- Molise
- Piedmont
- Autonomous Province of Bolzano
- Autonomous Province of Trento
- Apulia
- Sardinia
- Sicily
- Tuscany
- Umbria
- Aosta Valley
- Veneto
- To which age group do you belong?
- Up to 20 years old
- From 21 to 25 years old
- From 26 to 30 years old
- From 31 to 35 years old
- From 36 to 40 years old
- From 41 to 45 years old
- From 46 to 50 years old
- Over 50 years old
- What is your level of education?
- No educational qualification
- Elementary school certificate
- Middle school diploma
- Professional institute diploma
- High school diploma
- Bachelor’s degree
- Advanced studies, master’s, or postgraduate specialization
- Doctorate (PhD)
- Other

Please specify: ___________________

- In which of the following categories does your current profession fall into:

Please refer to the nomenclature and classification of ISTAT Professional Units available on the website <http://professioni.istat.it/sistemainformativoprofessioni/cp2011/>

- Students
- Unemployed
- Legislators, entrepreneurs, and senior management
- Intellectual, scientific, and highly specialized professions
- Technical professions
- Executive office work professions
- Commercial activities and services professions
- Artisans, skilled workers, and farmers
- Fixed and mobile machinery operators, and vehicle drivers
- Unqualified professions
- Homemakers
- Retirees
- Armed forces
- Other
- Do you have asthma?
- Yes
- No
- If yes, for how long?
- For less than 5 years
- From 5 to 10 years
- From 10 to 15 years
- Over 15 years
- What is the severity level of the illness?
  - Mild asthma
  - Moderate asthma
  - Severe asthma
- Do you regularly use digital devices such as PCs, tablets, smartphones, etc.?
  - Yes
  - No
- If you use them, what is the primary purpose?
- Work
- Leisure

## Section 2

DEFINITION

Digital Mindset refers to:

- A mental set made up of knowledge and experiences derived from living in a digitalized society, which are recognized and utilized by the individual in daily life.
- A mental attitude that is open and capable of recognizing the opportunities offered by digital transformation.
- An organizational behavior necessary to complement individual professional competencies.

Questions:

- How much do you agree with the following statement?

"I believe I have a digital mindset."

- - Strongly agree
  - Somewhat agree
  - Neither agree nor disagree
  - Somewhat disagree
  - Strongly disagree
- How much do you agree with the following statement?

"I believe I regularly use digital tools* for all the activities I perform at work, at home, at school, in social interactions, etc."

- - Strongly agree
  - Somewhat agree
  - Neither agree nor disagree
  - Somewhat disagree
  - Strongly disagree

* social networks, internet, PC, programs like Google Drive/TeamViewer, remote communication tools and/or streaming tools like Zoom/Microsoft Teams/Google Meet

- To what extent do you agree with the following statement?

“Regarding the management of my health, I believe I can use such digital skills or tools satisfactorily.”

- - Strongly agree
  - Somewhat agree
  - Neither agree nor disagree
  - Somewhat disagree
  - Strongly disagree
- In recent times, have you had more opportunities or have you been encouraged to use digital tools for managing your health?
  - Yes
  - No
- If so, which tools or initiatives promoted by patient associations and/or healthcare facilities have been encouraged?
- Initiatives for information about the pathology or disease management
- IT tools for assistance or support from the doctor
- IT tools for monitoring or detecting clinical parameters
- Telemedicine visits

(select the 2 most relevant)

- What was your level of satisfaction with this tool/initiative?
  - Completely satisfactory
  - Fairly satisfactory
  - Uncertain
  - Fairly unsatisfactory
  - Completely unsatisfactory

## Section 3

DEFINITION

According to the national guidelines from the Ministry of Health, Telemedicine refers to a mode of providing healthcare services through the use of innovative technologies, particularly Information and Communication Technologies (ICT), in situations where the healthcare professional and the patient (or two professionals) are not in the same location. Telemedicine involves the secure transmission of medical information and data in the form of texts, sounds, images, or other necessary formats for the prevention, diagnosis, treatment, and subsequent monitoring of patients.

Questions:

- When you need to communicate with your doctor, do you use digital tools such as the following?

|  | Yes | No | Occasionally |
| --- | --- | --- | --- |
| Telemedicine |  |  |  |
| Whatsapp/Telegram |  |  |  |
| E-mail |  |  |  |
| Social media |  |  |  |

- If you answered yes to the previous question, how satisfied are you with being able to use that tool?
  - Completely satisfied
  - Fairly satisfied
  - Uncertain
  - Fairly unsatisfied
  - Completely unsatisfied
- In the management of your health, do you think that the professional liability (civil and penal professional responsibilities) of the doctor are different if the visit is in person or if the following digital means are used?

|  | Yes | No |
| --- | --- | --- |
| Telemedicine |  |  |
| WhatsApp/Telegram |  |  |
| E-mail |  |  |
| Social media |  |  |

- If you answered yes, how concerned are you about using telemedicine as a tool?
- Very much
- A lot
- Quite a bit
- A little
- Not at all
- If you answered yes, how concerned are you about using WhatsApp/Telegram as a tool?
  - Very much
  - A lot
  - Quite a bit
  - A little
  - Not at all
- If you answered yes, how concerned are you about using e-mails as a tool?
  - Very much
  - A lot
  - Quite a bit
  - A little
  - Not at all
- If you answered yes, how concerned are you about using social media as a tool?
- Very much
- A lot
- Quite a bit
- A little
- Not at all
- Has your doctor ever recommended an app for use in healthcare?
- Yes
- No
- If yes, which one: ______________________(please specify)
- If yes, for which purpose?
- To remind me to take my medication
- To become increasingly aware of my illness and improve my understanding of the various aspects of the pathology
- To collect data to share with my doctor so they can understand if my illness is sufficiently controlled
- To participate in a clinical study or for clinical research purposes

(you can select more than one option)

- Has your doctor ever recommended websites for you to consult in order to gain more information and achieve greater awareness and self-management of your condition?
  - Yes
  - No
- If yes, which one/ones: _____________(please specify)
- Do you independently search for information on the internet before your specialist medical appointment?
  - Yes
  - No
- If yes, which are the online sources?
- WhatsApp/Telegram groups
- Social media
- Websites
- E-mail
- Others

Please specify: __________________

(you can select more than one option)

- Do you regularly use Telemedicine* for your specialist appointments or visits with your general practitioner?
- Yes
- No

*(refer to the above definition of Telemedicine)

- If so, in which of the following occasions?
  - Visits with specialist doctors
  - Visits with the general practitioner

(you can select more than one option)

- If so, for which diseases do you use it?
  - Asthma
  - Allergic rhinitis
  - Allergic conjunctivitis
  - Urticaria
  - Rhinosinusitis with or without nasal polyposis
  - Food allergy
  - Drug allergy
  - Other

Please specify: ________________-

(you can select more than one option)

- If you do not use it, what is the reason?
- I do not have access to such technology / connection at home or at work
- I don't know how to use the programs
- I don't trust it; I prefer to be examined in person
- My doctor has never suggested anything about it
- Have you ever used digital tools such as wearables, smart spirometry, or smart oximeters to collect data at home to send to your doctor?
  - Yes
  - No

If yes, please specify which ones: _____________

- If you answered no, what do you think are the most limiting factors?
- Lack of familiarity with these digital tools
- Lack of trust from the patient in using this equipment
- Little confidence in the usefulness of such tools on the part of the doctor, who advises against their use
- Difficulty obtaining the equipment and/or covering the costs personally

(select up to 3 options)

## Section 4

Questions:

- Regarding inhalation therapies, do you know what a Smart Inhaler is and/or have you ever had the opportunity to use one?
- I know what it means and have had the opportunity to use it in my clinical practice
- I know what it means but have never had the opportunity to use it
- It’s not entirely clear to me what it means

DEFINITION:

A Smart Inhaler consists of a digital sensor applied to an inhaler that connects to a patient’s smartphone app and allows for a "two-way data exchange" (e.g., inhalation times and methods, air quality, self-assessment of asthma control, etc.).

Questions:

- Given the definition above, what benefits do you think could result from associating an inhaler with a digital support?
- It would definitely be helpful to remind me to take the prescribed therapy through audio and video notifications
- It could help me manage my condition better by alerting me if I’m about to have a flare-up
- It could be useful to get feedback on correct inhalation technique and information about temperature and air quality
- It would not bring me any benefit in managing my condition
- It would be too complicated for daily use

(select up to 3 options)

- If you also selected also the last answer, what do you think are the main limiting factors?
  - Insufficient personal knowledge of digital technologies to be able to use such tools
  - Low willingness of specialist doctors to prescribe this type of support
  - Low willingness of general practitioners and pharmacists to provide support for the operation and proper use of these devices
  - Lack of time to use such devices during the day

(select up to 3 options)

- Which interlocutors should be contacted for inhalation therapies with digital support to be effective for you?
- Myself, who, with proper initial explanation from my doctor, could manage the therapy even with digital support
- My family members (parents, children, siblings, partner), whom I would like to have the functioning of these tools explained to
- The professionals who help me manage my asthma

(you can select more than one option)

- If you also selected the last answer, which of the following professional figures would you definitely want to be involved?
- The specialist doctor
- The pharmacist
- The nurse from the department/clinic where specialist follow-up visits are conducted
- The general practitioner

(you can select more than one option)

## Section 5

DEFINITION

Digital Therapies* are therapeutic interventions mediated by software, designed for a specific disease, and aimed at modifying a patient's behavior to improve the outcomes of their condition. These digital therapeutic interventions must be developed through randomized controlled clinical trials, meaning they must be based on clear clinical evidence of efficacy.

The active component is the therapeutic algorithm, which is the element responsible for the clinical effect; the excipient is an aspect of the software that makes the treatment as digitally bioavailable as possible (e.g., modules for rewarding the patient, gamification, reminders for taking digital therapy and complementary therapies, modules to connect the patient with their doctor and with other patients with the same condition, etc.).

- Based on the definition, do you believe that the doctors treating you for your respiratory condition are sufficiently informed about it?
  - Strongly agree
  - Somewhat agree
  - Neither agree nor disagree
  - Somewhat disagree
  - Strongly disagree
- Given the definition, do you believe that your level of knowledge of Digital Therapies is adequate?
  - Strongly agree
  - Somewhat agree
  - Neither agree nor disagree
  - Somewhat disagree
  - Strongly disagree

Comments
