# Supplementary material for: Digital Innovation in Asthma Management in Italy: Results From the “Confronting Asthma Survey”
Source: Clin Transl Allergy. 2025 Oct 17;15(10):e70109. doi: 10.1002/clt2.70109 (PMC12533498; doi:10.1002/clt2.70109)
Supplement: Supplementary file 2 — Supporting Information S2 [file CLT2-15-e70109-s002.docx]

# DIGITAL INNOVATION SURVEY – FOR PHYSISCIANS

Dear Participant,

We thank you in advance for your availability and interest in this research, which aims to gather information about knowledge and attitudes towards the use of digital health tools in the healthcare field.

Our goal is to describe the technological means used by patients and doctors to communicate with one another, clarify the types of content exchanged, and obtain an assessment of the quality and effectiveness of digital communication.

The completion of the questionnaire will take approximately 10 minutes, but you are free to discontinue at any moment.

We guarantee that all collected data will be treated with the utmost confidentiality and privacy, will be analyzed anonymously, and used exclusively for research purposes.

By selecting "Confirm," you declare that you have understood the purpose of the study and authorize the processing of personal data for research activities in an anonymous form.

- Confirm
- Do not confirm

## Section 1

Questions:

- In which region do you practice?
  - Abruzzo
  - Basilicata
  - Calabria
  - Campania
  - Emilia Romagna
  - Friuli Venezia Giulia
  - Lazio
  - Liguria
  - Lombardy
  - Marche
  - Molise
  - Piedmont
  - Autonomous Province of Bolzano
  - Autonomous Province of Trento
  - Apulia
  - Sardinia
  - Sicily
  - Tuscany
  - Umbria
  - Aosta Valley
  - Veneto
- What is your specialization?
- Pulmonologist
- Allergist
- Other

Please specify: _____________________

- In what type of facility do you currently work?

- Hospital
- University hospital
- Territorial outpatient clinic

(you can select more than one option)

- Do you regularly use digital devices such as PCs, tablets, smartphones, etc.?
- Yes
- No
- If you use them, what is the primary purpose?
- Work
- Leisure

## Section 2

DEFINITION

Digital Mindset refers to:

- A mental set made up of knowledge and experiences derived from living in a digitalized society, which are recognized and utilized by the individual in daily life.
- A mental attitude that is open and capable of recognizing the opportunities offered by digital transformation.
- An organizational behavior necessary to complement individual professional competencies.

Questions:

- How much do you agree with the following statement?

"I believe I have a digital mindset."

- Strongly agree
- Somewhat agree
- Neither agree nor disagree
- Somewhat disagree
- Strongly disagree
- How much do you agree with the following statement?

"I believe I regularly use digital tools* for all the activities I perform at work, at home, at school, in social interactions, etc."

- - Strongly agree
  - Somewhat agree
  - Neither agree nor disagree
  - Somewhat disagree
  - Strongly disagree

* (social networks, internet, PC, programs like Google Drive/TeamViewer, remote communication tools and/or streaming tools like Zoom/Microsoft Teams/Google Meet)

- To what extent do you agree with the following statement?

"In the workplace, I believe I can make satisfactory use of my knowledge of digital technologies and digital health support tools."

- - Strongly agree
  - Somewhat agree
  - Neither agree nor disagree
  - Somewhat disagree
  - Strongly disagree
- Has the company or organization you work for promoted initiatives specifically related to digital health?
- Yes
- No
- If so, what type of initiative has been promoted?
- Training initiatives
- Informatic tools to support medical activities
- Research initiatives
- IT tools to support patients

(please select the 2 most relevant)

- What was your level of satisfaction?
- Completely satisfactory
- Fairly satisfactory
- Uncertain
- Fairly unsatisfactory
- Completely unsatisfactory

## Section 3

DEFINITION

According to the national guidelines from the Ministry of Health, Telemedicine refers to a mode of providing healthcare services through the use of innovative technologies, particularly Information and Communication Technologies (ICT), in situations where the healthcare professional and the patient (or two professionals) are not in the same location. Telemedicine involves the secure transmission of medical information and data in the form of texts, sounds, images, or other necessary formats for the prevention, diagnosis, treatment, and subsequent monitoring of patients.

Questions:

- When you need to communicate with a patient, do you use digital tools such as the following?

|  | Yes | No | Occasionally |
| --- | --- | --- | --- |
| Telemedicine |  |  |  |
| WhatsApp/Telegram |  |  |  |
| E-mail |  |  |  |
| Social |  |  |  |

- If you answered yes to the previous question, how satisfied are you with being able to use that tool?
  - Completely satisfied
  - Fairly satisfied
  - Uncertain
  - Fairly unsatisfied
  - Completely unsatisfied
- Do you have any concerns or doubts related to the current regulations on professional liability (civil and penal responsibility) when using any of these digital tools?

|  | Yes | No |
| --- | --- | --- |
| Telemedicine |  |  |
| WhatsApp/Telegram |  |  |
| E-mail |  |  |
| Social media |  |  |

- If you answered yes, how concerned are you about using telemedicine as a tool?
- Very much
- A lot
- Quite a bit
- A little
- Not at all
- If you answered yes, how concerned are you about using WhatsApp/Telegram as a tool?
  - Very much
  - A lot
  - Quite a bit
  - A little
  - Not at all
- If you answered yes, how concerned are you about using e-mails as a tool?
  - Very much
  - A lot
  - Quite a bit
  - A little
  - Not at all
- If you answered yes, how concerned are you about using social media as a tool?
- Very much
- A lot
- Quite a bit
- A little
- Not at all
- Have you ever recommended a healthcare-related app to your patients?
- Yes
- No
- If yes, which one/ones: ___________________(please specify)
- If yes, which was the purpose?
- To improve adherence to therapy
- To improve disease awareness and understanding of one’s own medical condition
- To collect data to share with the individual patient in order to enhance clinical management
- To collect data for trials, Real World Evidence

(you can select more than one option)

- Have you ever recommended, or do you routinely recommend, that your patients consult websites to improve adherence and awareness of their condition?
  - Yes
  - No
- If yes, which one/ones? ____________________(please specify)
- Do your patients tend to search for information on the internet on their own before a specialist medical consultation?
- Yes
- No
- If yes, which online sources do they use?
- WhatsApp/Telegram groups
- Social media
- Websites
- E-mail
- Other

Please specify: _______________________________

(you can select more than one option)

- Do you regularly use Telemedicine* in managing your patients?
- Yes
- No

(*Refer to the above definition of Telemedicine)

- If so, in which of the following occasions?
  - First visit
  - Follow-up visit of a previously known patient
  - Only for specific diseases

(you can select more than one option)

- If so, for which diseases do you use it?
  - Asthma
  - Allergic rhinitis
  - Allergic conjunctivitis
  - Urticaria
  - Rhinosinusitis with or without nasal polyposis
  - Food allergy
  - Drug allergy

(you can select more than one option)

- If you do not use it, what is the reason?
  - There are IT infrastructure issues in my organization
  - I am not able to use the programs
  - I don’t trust it; I prefer to see the patient in person
  - I am concerned about issues related to professional responsibility

(you can select more than one option)

- Have you ever used digital tools such as wearables, smart spirometers, or smart oximeters to collect data from your patients at home?
  - Yes
  - No
- If so, please specify which ones: ____________________
- If you answered no, what do you think are the most limiting factors?
- Lack of familiarity with these digital tools
- Lack of trust from the patient in using this equipment
- Absence of clear regulations regarding the use of these tools in the context of a clinical visit or patient monitoring
- Difficulty for patients to obtain the equipment or cover the costs themselves

(Select up to 3 options)

## Section 4

Questions:

- Regarding inhalation therapies, do you know what a Smart Inhaler is and/or have you ever had the opportunity to use one?
- I know what it means and have had the opportunity to use it in my clinical practice
- I know what it means but have never had the opportunity to use it
- It’s not entirely clear to me what it means

DEFINITION:

A Smart Inhaler* consists of a digital sensor applied to an inhaler that connects to a patient’s smartphone app and allows for a "two-way data exchange" (e.g., inhalation times and methods, air quality, self-assessment of asthma control, etc.).

Questions:

- Given the definition*, what benefits do you think could result from associating an inhaler with a digital support?
- It could improve patient adherence and reduce the occurrence of side effects related to incorrect or excessive use of the medication
- It could be beneficial for patient management by making them more aware of their condition and helping them recognize when an exacerbation is about to occur
- It could be useful for collecting data on patient adherence to treatment and inhalation techniques
- It would likely not be appreciated by the patient
- In clinical practice, it would be difficult to apply, and its use could be limited by multiple factors

(Select up to 3 options)

- If you also selected also the last answer, what do you think are the main limiting factors?
- The lack of adequate digital skills on my part and that of my healthcare collaborators (doctors, nurses)
- The lack of digital skills among healthcare workers in the community who will be responsible for therapeutic continuity (general practitioners, pharmacists)
- The lack of adequate digital skills among patients
- Insufficient time during a medical visit to explain the usefulness of the inhaler and how to use it
- Lack of time to review the data generated

(Select up to 3 options)

Which interlocutors should be contacted for inhalation therapies with digital support to be effective for the patient?

- The patient, who with proper education on usage can manage the digital therapy on their own
- The caregivers (parents, children, siblings, partner)
- The general practitioner
- The specialist doctor and/or the nurses in the clinic/ward
- The pharmacists

(Select up to 3 options)

## Section 5

DEFINITION

Digital Therapies* are therapeutic interventions mediated by software, designed for a specific disease, and aimed at modifying a patient's behavior to improve the outcomes of their condition. These digital therapeutic interventions must be developed through randomized controlled clinical trials, meaning they must be based on clear clinical evidence of efficacy.

The active component is the therapeutic algorithm, which is the element responsible for the clinical effect; the excipient is an aspect of the software that makes the treatment as digitally bioavailable as possible (e.g., modules for rewarding the patient, gamification, reminders for taking digital therapy and complementary therapies, modules to connect the patient with their doctor and with other patients with the same condition, etc.).

Questions:

- Given the definition*, do you believe that the level of knowledge of Digital Therapies within your field is adequately understood?
  - Strongly agree
  - Somewhat agree
  - Neither agree nor disagree
  - Somewhat disagree
  - Strongly disagree

(*Refer to the definition above)

- Given the definition*, do you believe that your level of knowledge of Digital Therapies is adequate?
  - Strongly agree
  - Somewhat agree
  - Neither agree nor disagree
  - Somewhat disagree
  - Strongly disagree

(*Refer to the definition above)

Comments
