# Supplementary material for: Digital Innovation in Asthma Management in Italy: Results From the “Confronting Asthma Survey”
Source: Clin Transl Allergy. 2025 Oct 17;15(10):e70109. doi: 10.1002/clt2.70109 (PMC12533498; doi:10.1002/clt2.70109)
Supplement: Supplementary file 3 — Supporting Information S3 [file CLT2-15-e70109-s001.docx]

**Table 1S.** Characteristics and perspectives of doctors and asthmatic patients (Survey results).

| **Variable / Category** | **Doctors**  **(n=180)** | **Asthmatic patients (n=154)** |
| --- | --- | --- |
| ***Characteristics of doctors surveyed*** | | |
| **Medical specialty** | **Doctors (n= 158)** |  |
| Pulmonology | 68 (43.0%) |  |
| Allergology | 63 (39.9%) |  |
| Other* | 27 (17.1%) |  |
| **Healthcare setting** | **Doctors (n=175)** |  |
| Hospital-based | 85 (47.2%) |  |
| University-affiliated | 70 (38.9%) |  |
| Territorial outpatient clinic | 20 (11.1%) |  |
| ***Characteristics of asthmatic patients surveyed*** | | |
| **Sex** |  | **Patients (n=134)** |
| Male |  | 39 (29.1%) |
| Female |  | 95 (70.9%) |
| **Age distribution** |  | **Patients (n=134)** |
| Under 30 years |  | 11 (7.9%) |
| Between 30 and 50 years |  | 51 (38.3%) |
| Over 50 years |  | 72 (53.7%) |
| **Education** |  | **Patients (n=134)** |
| Primary school diploma |  | 4 (3.0%) |
| High school diploma |  | 50 (37.3%) |
| Bachelor's/Master’s degree |  | 32 (23.9%) |
| Postgraduate/Master’s specialization |  | 19 (14.2%) |
| PhD |  | 2 (1.5%) |
| **Occupation** |  | **Patients (n=134)** |
| Students |  | 7 (5.2%) |
| Unemployed and homemakers |  | 12 (9.0%) |
| Executives and intellectual professions |  | 23 (17.2%) |
| Technical and commercial office professions |  | 28 (20.9%) |
| Artisans, technical professions, and armed forces |  | 12 (9.0%) |
| Low-skilled occupations |  | 3 (2.2%) |
| Retired |  | 19 (14.2%) |
| Other |  | 30 (22.4%) |
| ***Characteristics of doctors and asthmatic patients surveyed*** | | |
| **Geographical distribution** | **Doctors (n=161)** | **Patients (n=134)** |
| Northern Italy | 51 (31.7%) | 40 (29.9%) |
| Central Italy | 59 (36.6%) | 83 (61.9%) |
| Southern Italy | 51 (31.7%) | 11 (8.2%) |
| **Habitual use of digital tools** | **Doctors (n=160)** | **Patients (n=133)** |
| Use of digital tools | 159 (99.4%) | 123 (92.5%) |
| For work | 137 (86.2%) | 69 (57.0%) |
| For leisure | 22 (13.8%) | 52 (43.0%) |
| ***Perspectives of doctors and asthmatic patients surveyed*** | | |
| **Patient-doctor digital communication tools**** | **Doctors (n=180)** | **Patients (n=154)** |
| Telemedicine | 47 (26.1%) | 65 (42.2%) |
| WhatsApp/Telegram | 43 (23.9%) | 43 (27.9%) |
| E-mail | 38 (21.1%) | 36 (23.4%) |
| Social media | 51 (28.3%) | 65 (42.2%) |
| **Online sources consulted before visiting doctor** | **Doctors (n=110)** | **Patients (n=73)** |
| WhatsApp/Telegram | 20 (18.2%) | 6 (8.2%) |
| Social media | 46 (41.8%) | 28 (38.4%) |
| Websites | 99 (90.0%) | 66 (90.4%) |
| E-mail | 3 (2.7%) | 0 (0.0%) |
| Other*** | 2 (1.8%) | 11 (15.1%) |
| **Perceived benefits of smart inhalers** | **Doctors (n=130)** | **Patients (n=114)** |
| Reminder for therapy | 93 (71.5%) | 52 (45.6%) |
| Managing/preventing exacerbations | 61 (46.9%) | 59 (51.8%) |
| Support for inhalation technique | 79 (60.8%) | 58 (50.9%) |
| **Barriers to use of smart inhalers†** | **Doctors (n= 11)** | **Patients (n=17)** |
| Poor digital skills – healthcare providers | 2 (18.2%) | 3 (17.6%) |
| Poor digital skills – GPs/pharmacists | 4 (36.4%) | 5 (29.4%) |
| Poor digital skills – patients | 8 (72.7%) | 6 (35.3%) |
| Lack of time | 6 (54.5%) | 7 (41.2%) |

* Other specialties include internal medicine and pediatrics.

** Referred to the tools actually used in patient-doctor communication.

*** Other sources include forums, patient associations, institutional websites.

**†** Percentages based on respondents who reported barriers to using smart inhalers.

**Table 2S.** Attitudes toward digital technologies of the surveyed doctors according to their medical specialty and the asthmatic patients stratified by age.

| ***DOCTORS*** | **1**  **Pulmonologist (n=68)** | **2**  **Allergologist**  **(n=63)** | **3**  **Others***  **(n=27)** | **p value** |
| --- | --- | --- | --- | --- |
| Habitual use of digital tools | 68 (100%) | 63 (100%) | 27 (100%) | N/A |
| Telemedicine use | 12 (17.6%) | 14 (22.2%) | 5 (18.5%) | 0.880 |
| Use of wearables and smart devices | 14 (20.6%) | 8 (12.7%) | 5 (18.5%) | 0.585 |
| Awareness and use of smart inhalers | 21 (30.9%) | 19 (30.2%) | 4 (14.8%) | 0.263 |
| Recommendation of health-promoting apps | 17 (25.0%) | 26 (41.3%) | 7 (25.9%) | **0.048** |

* Other specialists include internal medicine physicians and pediatricians.

| ***ASTHMATIC PATIENTS*** | **1**  **Under 30 years (n=11)** | **2**  **30 - 50 years (n=51)** | **3**  **Over 50 years (n=72)** | **p value** |
| --- | --- | --- | --- | --- |
| Habitual use of digital tools | 11 (100%) | 49 (96.1%) | 61 (84.7%) | 0.057 |
| Telemedicine use | 1 (9.1%) | 4 (7.8%) | 3 (4.2%) | 0.781 |
| Use of wearables and smart devices | 2 (18.2%) | 3 (5.9%) | 7 (9.7%) | 0.430 |
| Awareness and use of smart inhalers | 1 (9.1%) | 4 (7.8%) | 1 (1.4%) | 0.088 |
| Recommendation of health-promoting apps | 2 (18.2%) | 3 (5.9%) | 4 (5.5%) | 0.330 |
